# 3’UTR regulation of axon translation and optic nerve regeneration

**DOI:** 10.1186/s13024-026-00952-2

**Published:** 2026-05-29

**Authors:** Minjuan Bian, Emma L. Huie, Xin Xia, Michael Nahmou, Savana A. Maxfield, Liang Li, Liang Liu, Ziming Luo, Christina Tsien, Katherine J. Healzer, Heather V. Chang, Kristina Russano, Jeffrey L. Goldberg

**Affiliations:** Department of Ophthalmology, Mary M. and Sash A. Spencer Center for Vision Research, Byers Eye Institute, Stanford University School of Medicine, Palo Alto, CA 94304 USA

**Keywords:** Local translation, Axon regeneration, 3’UTR, FRAP, AAV, Trophic factors, Forskolin, Src, FAK

## Abstract

**Background:**

In the adult mammalian central nervous system (CNS), failure of axon regeneration limits recovery after traumatic injury or in neurodegenerative disease. Local protein translation has been implicated in the regulation of axon growth in highly compartmentalized neurons. 3’ untranslated regions (3’UTRs) of mRNAs play critical roles in RNA localization and modification. Here we studied the regulation of 3’UTRs in growth cone and axon regeneration.

**Methods:**

Using fluorescence recovery after photobleaching (FRAP), we examined dynamic changes of mRNA 3’UTRs-related local translation in distal growth cones of primary neurons, initially comparing 3’UTRs from Gap43, normally localized to axons, and gamma-actin (Actg1), normally distributed to soma and axon. Local translation patterns in response to trophic factors and depolarization stimuli were analyzed, with or without translation inhibitor anisomycin. Adeno-associated viral vectors were used to express constitutively active Src with specific 3’UTRs after optic nerve injury in vivo. Axon growth and Src signaling were detected to identify function of 3’UTRs in axon regeneration. Statistical analysis was performed using one-way ANOVA or Kruskal-Wallis test, two-tailed unpaired t-test or Mann-Whitney test for data sets with different distributions.

**Results:**

We discovered different 3’UTRs led to differences in local translational regulation in growth cones in vitro, including in response to relevant signals such as brain-derived neurotrophic factor (BDNF), forskolin, ciliary neurotrophic factor (CNTF), depolarization and repolarization. In vivo, we found that addition of 3’UTRs from Gap43 or Actg1 to a construct expressing constitutively active Src, which normally regulates growth cone and axon elongation, increased Src mRNA and protein localization, and Src activity measured by phospho-FAK in the optic nerve and optic tract, and the 3’UTR of Gap43 promoted more long-distance axon regeneration after optic nerve injury.

**Conclusions:**

Together these data enhance our understanding of the complexity of 3’UTR-mediated regulation of local axon translation, and point to potential therapeutic avenues for growth cone-related protein expression and local translation via 3’UTR manipulation.

## Background

Central nervous system (CNS) axons in adult mammals fail to regenerate after injury or in neurodegenerative disease. Because of the relatively simple anatomy and accessibility, retinal ganglion cells (RGCs) and the optic nerve in the visual system have been widely used to study neuronal survival and axon regeneration in the CNS. Optic nerve axons are highly vulnerable to injury in trauma, ischemia, and glaucoma, among others. In all of these optic neuropathies, the progressive degeneration of optic nerve axons and subsequent RGC death cause irreversible vision loss in patients [1, 2]. Some axonal regeneration can be elicited by various treatments including targeting transcription factors such as KLF9 deletion and Lhx2 overexpression [3, 4]; signaling pathways such as activating Akt/mTOR via Pten knockout [5], osteopontin overexpression [6], Akt overexpression [7], activating immune system such as with zymosan [8] or curdlan [9]; neurotrophic factors such as ciliary neurotrophic factor (CNTF) [10], cyclic adenosine monophosphate (cAMP) [11], or Lin28a overexpression [12, 13]. Axon regeneration can also be enhanced by modulating axon-specific factors targeting cytoskeleton as with myosin IIA/B deletion [14] or mitochondrial transport [15], but relatively little is known about how axon growth is regulated locally in axons [16].

Although these and other genes have been studied for their function in promoting axon regeneration, little is known about how their localization or delivery to axons regulates these functions. RNA localization and translation in sub-compartments of cells play critical roles in regulating spatial and temporal gene expression patterns during various cellular events [17, 18], especially in highly polarized neurons [19–28]. mRNA transport into axons, which are equipped with translation machinery, contributes to neuronal functions such as axon guidance, synaptogenesis, branching, and response to axonal injury, allowing growth cones and axon terminals to act precisely and rapidly to stimuli even at long distances from the cell body [23, 27, 29, 30]. Recent advances in deep sequencing have revealed the localization of thousands of mRNAs in axons, pointing to the potential importance of local regulation of translation [20, 31]. 3’ untranslated regions (3’UTRs) of mRNAs have been implicated in multiple studies to specify RNA localization and regulate post-translational modification [27, 32, 33]. Emerging evidence has indicated the important roles of 3’UTRs in promoting axon growth in the peripheral nerve system (PNS) by facilitating axonal transport or local translation of target genes essential for regeneration [34, 35].

The functions of 3’UTRs in CNS neurons, however, have not been explored, and for proteins that may act in the axon to promote regeneration, it’s not known to what degree regeneration would be facilitated by increasing axon transport of mRNAs and local translation, or whether trophic factors that promote axon growth act on mRNA translation. Here we used fluorescence recovery after photobleaching (FRAP) to examine new translation of mRNA in growth cones of primary neurons in vitro, comparing an example 3’UTR from a gene localized to axons, Gap43, and an example 3’UTR from a gene with soma and axon distribution, Actg1. Extending these results in vivo, we further explore the potential for 3’UTR-mediated axon delivery of a growth cone-associated signaling protein, constitutively active Src, to enhance RGC axon regeneration after optic nerve injury.

## Methods

### Animals and ethics

All use of animals conformed to the ARVO Statement for the Use of Animal in Ophthalmic and Vision Research, all animal procedures were approved by the Administrative Panel of Laboratory Animal Care (APLAC) and the Institutional Animal Care and Use Committee (IACUC) at Stanford University. Wild type C57BL/6 mice were obtained from Charles River. Wild type 129 × 1/Svj mice were obtained from Jackson Laboratory.

### 3’UTR constructs, viral plasmids and AAV packaging

Photoswitchable Dendra2 with myristoylation element (^myr^Dendra) was used in all of the fluorescent reporter plasmids for local translation analysis [36, 37]. 40 nucleotides (nt) of Gap43 3’UTR [38]; full length of Actg1 3’UTR; or 51 nt of Nrgn 3’UTR [39] were synthesized and cloned directly into p^myr^Dendra backbone using Gibson Assembly (New England Biolabs, E5510S).

The coding region of mouse Src with constitutive active mutation in the kinase domain (Y527F) followed by the 3’Gap43 or 3’Actg1 UTRs as above were synthesized and subcloned into previously described backbone pAM-AAV-mSncg-3HA-WPRE [40] with XbaI, SacI or HpaI sites; Src-Y527F without 3’UTR served as a control. As a negative control, luciferase was synthesized and subcloned into the same AAV backbone. Flag-tag sequence was added to the N-terminus of Src and luciferase constructs. The protocol for production of triple mutant AAV2 (Y444, 500, 730 F) has been described previously [40, 41]; real-time PCR was performed to check AAV titers.

### Primary hippocampal neuron culture and transfection

Hippocampal neurons were isolated from C57BL/6 embryonic day 18 embryos as previously described with minor modification [42]. Briefly, mouse hippocampus was dissected in ice cold HBSS without calcium and magnesium and digested with 0.05% trypsin-EDTA in phosphate-buffered saline (PBS) for 30 min at 37 °C. The dissociated tissue was then pelleted and treated with DNase (Worthington Biochemical, LS002006) in HBSS with calcium and magnesium. After two washes and spin centrifugations, neurons were dissociated in Neurobasal (Thermo Fisher Scientific, 21103049) supplemented with 2mM L-Glutamine (Thermo Fisher Scientific, 25030081), 2% B-27 (Thermo Fisher Scientific, A3582801) and 2.5% fetal bovine serum (Thermo Fisher Scientific, 10437028), then were plated on 4 well glass-bottom Lab-Tek chamber slides (Thermo Fisher Scientific, 155382) pre-coated with poly-D-lysine (Sigma-Aldrich, P0899). The medium was replaced with fresh medium without fetal bovine serum after overnight incubation. Two days later, neurons were transfected with different 3’UTR constructs individually using Lipofectamine LTX with PLUS Reagent (Invitrogen, 15338-100). Neurons were allowed to be cultured for one additional day before live cell imaging.

### FRAP recording and pharmacological treatments

FRAP was performed on primary hippocampal neurons transfected with ^myr^Dendra constructs. Cells were maintained with 5% CO_2_ at 37 °C during imaging. 405 nm laser on confocal laser scanning microscope (Zeiss, LSM 880) was used to bleach Dendra signals (Laser power 15%, 8–10 cycles with 3 ms interval). Prior to photobleaching, distal growth cone regions were imaged with 488 nm and 555 nm laser lines to obtain baseline fluorescence signals. The same excitation and emission parameters were used to acquire post-photosbleaching images at 0 min, 20 min and 40 min.

Distal growth cones that were farther than 80 μm away from soma were recorded and set as regions of interest (ROI). Fluorescence intensities in the ROIs were calculated by Zeiss software. For normalization across experiments, the fluorescence intensities at 488 nm at t = 0 min (post-bleach) was set as 0% and subtracted from intensity values at 20–40 min; the percentage of intensity recovery at each time point was normalized to the pre-bleach intensity (set as 100%) for data analysis. ROIs with decreased fluorescent intensity at 555 nm were excluded from quantification.

**Fig. 1:**
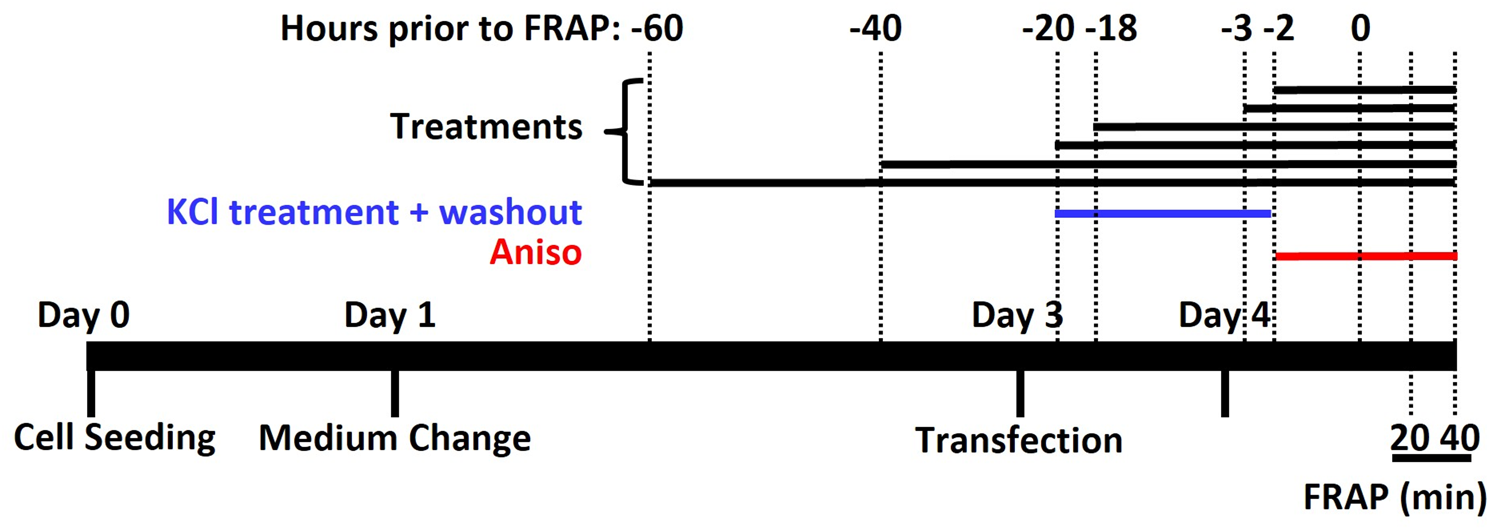
Timeline of primary hippocampal neuron culture, pharmacological treatments and FRAP recording

Treatments with BDNF (50 ng/ml, Thermo Fisher Scientific, 450-02), forskolin (5 µM, Sigma-Aldrich, F6886), CNTF (50 ng/ml, Thermo Fisher Scientific, 450 − 13), Rho-associated kinase inhibitor (ROCKi) (10 µM, STEMCELL, Y-27632), KCl (40 mM), or calcium channel agonist Bay K 8644 (3 µM, Sigma-Aldrich, B112) were applied in the 2 through 60 h preceding the FRAP assays, as marked in Fig. 1. Anisomycin (20 µM, Sigma-Aldrich, A9789) was administrated 2 h prior to FRAP. The hippocampal neurons were pre-conditioned with L-type calcium channel blocker nifedipine (10 µM, Sigma-Aldrich, N7634) or N-type calcium channel blocker MVIIA (300 nM, Sigma-Aldrich, C1182) for 2 h, followed by KCl treatment for 18 h prior to FRAP. For the KCl washout, medium was replaced with fresh medium without KCl for 2 h before FRAP recording.

### DiBAC staining

Primary hippocampal neuron was treated with KCl for 2 h, 20 h, 18 h then washout for 2 h or NaCl for 20 h, respectively. Membrane potential dye DiBAC_4_(3) (10µM, Thermo Fisher Scientific, B438) was then added to culture medium 1 h prior to live cell imaging. All conditions were imaged at the same time using the same parameter setting on a confocal laser scanning microscope (Zeiss, LSM 880). 488 nm laser and bright field images were captured on the same field.

### Intravitreal injection of AAVs and anterograde labeling

AAVs were injected intravitreally through the sclera 1 mm behind the limbus by foot-pedal controlled Picospritzer III-Intracellular Microinjection Dispense Systems (Parker Co.) under isoflurane anesthesia. AAVs were diluted with Pluronic F-68 (Sigma-Aldrich, P1300), approximately 7×E10-1×E11 viral genome/eye of AAVs were applied to each eye in the volume of 1.25 µl. For anterograde labelling of regenerating axons, 1 µl of cholera toxin subunit B (CTB555, Invitrogen, C22843) at 1 µg/µl in PBS was intravitreally injected to each eye 2 days prior to tissue harvest.

### Optic nerve crush mouse model

Optic nerve crush (ONC) was performed two weeks after the intravitreal injection of AAVs as described previously [43]. After anesthesia, optic nerve was exposed from lateral canthus and ONC was then performed using cross-action forceps (Roboz Surgical Instrument) for 3 s at 2–3 mm behind the globe. Care was taken to avoid impairing blood supply to the retina.

### Sample preparation and immunostaining

All mice were deeply anesthetized, perfused with PBS and then 4% paraformaldehyde (PFA) in PBS. Eyes, optic nerves and brains were dissected carefully, post-fixed with 4% PFA in PBS for 1–2 h and washed with PBS. The optic nerves and brains from 2 weeks post intravitreal injection and optic nerves from 2 weeks after ONC (2wpc) were collected and processed for cryosectioning, to quantify CTB-555 positive axons, as well as for immunofluorescence, and in situ hybridization (BaseScope) using the same set of tissues. Samples were processed with sequential treatment of 15% sucrose and 30% sucrose in PBS, then embedded with optimal cutting temperature (OCT) for frozen sectioning on a Leica cryostat. Optic nerve sections with a thickness of 14 μm and brain sections with a thickness of 20 μm were collected.

For immunostaining, sections were blocked and permeabilized with 10% normal goat serum and 0.3% Triton in PBS for 1 h at room temperature, then labeled with anti-p-Focal Adhesion Kinase (FAK)-Y397 (1:250, Abcam, ab81298) in blocking buffer overnight at 4 °C. The following day, sections were washed with PBS, then incubated with Alexa Fluor 647 conjugated goat anti-rabbit IgG (1:1000, ThermoFisher Scientific, A-21245) in PBS for 1 h at room temperature. Followed by three PBS washes, sections were mounted with ProLong Gold Anti-Fade mounting medium (ThermoFisher Scientific, F36934). Images were taken under confocal laser scanning microscope (Zeiss, LSM 880) with the same parameter settings.

The quantification of positive pFAK-Y397 signals from optic nerve and optic tract was performed by investigators masked to conditions. Three ROIs (100 μm x 100 μm), located at 0, 500 and 1000 μm from optic chiasm, were selected from the maximum intensity projection of wholemount optic nerve images. Mean fluorescence intensity was measured after thresholding using Fiji, and then normalized to the luciferase group from each data set for multiple comparison. Results were presented as normalized pFAK-Y397 intensity value per optic nerve for each sample. ROIs for pFAK-Y397 signal quantification were defined on CTB555 channel in brain sections containing optic tract. Following thresholding in Fiji, the number and area of positive pFAK-Y397 particles were counted within each ROI. The normalized density of pFAK-Y397-positive particles was then calculated for data analysis.

### Retinal flatmount immunofluorescence and quantification of RGC survival

Retinas were dissected out from eyes and blocked with 10% goat serum and 2% Triton in PBS, then immunostained with anti-RNA-binding protein with multiple splicing (RBPMS) (1:2000, custom made, guinea pig, ProSci Co.) or anti-Flag(M2) (1:300, mouse, Sigma-Aldrich, F1804) with overnight incubation at 4 °C. After washing with PBS, Alexa Fluro 647 goat anti-guinea pig IgG (1:200, ThermoFisher Scientific, A21450), or Alexa Fluro 405 goat anti-mouse IgG (1:500, ThermoFisher Scientific, A31553) in PBS were applied for 1–2 h of incubation at room temperature. Samples were washed with PBS, then mounted with ProLong Gold Anti-Fade mounting medium, imaged under the same microscope mentioned above.

The quantification of RGC survival was performed by investigators masked to conditions. 8 fields (210 μm x 210 μm) from 4 flatmount quadrants were imaged, 1200–1300 μm and 1800–1900 μm away from optic nerve head was set as middle and periphery regions, respectively. RBPMS positive cells were counted using RGCode [44] with minor adjustments; results were presented as cells/mm^2^ for each sample.

### Optic nerve processing and quantification of axon growth

Optic nerve regeneration in the 2-week samples was quantified using longitudinal sections of optic nerve. 6–8 continuous sections, each with the thickness of 14 μm, were collected and imaged on a confocal laser scanning microscope with a 25x oil objective (Zeiss, LSM 880), using the interval of 2 μm for tiling of wholemount optic nerve. The number of CTB-positive axons from each maximum projection image that crossed 250–4000 μm from the crush site were counted every 250 μm in a masked fashion. The width of each section were measured as W and the radius (r) of the optic nerve was set from widest W/2. Axon density at each distance was estimated as axon numbers divided by W*14 µm. The final estimated axon number per optic nerve was then calculated by multiplying axon density by cross-sectional area of axon ($\:A=\pi\:{r}^{2}$). The logarithm(base 10) of estimated axon numbers was plotted; samples without axons were not displayed as dots. Estimated axon totals or actual axon numbers were compared across experimental groups.

Because we were concerned about missing rare axons in the longer time point given ongoing RGC death after optic nerve crush, optic nerve samples from 6 weeks after optic nerve crush (6wpc) were whole-mount imaged after clearing with the iDISCO method [45] with minor modifications [41]. Optic nerve samples were kept in PBS until sequential treatments with 40%, 80%, 100%, 100% methanol in H_2_O for 40 min at each concentration, then dichloromethane (DCM) / methanol (2:1) for 30 min, 100% DCM for 30 min, and final dibenzyl ether (DBE) for 1 min. Cleared optic nerve samples were mounted in DBE for whole optic nerve imaging. Cleared optic nerves were imaged on confocal laser scanning microscope at a 25x oil objective (Zeiss, LSM 880). The multiple optical sliced images covering whole optic nerve including the crush site and chiasm were collected and tiled after acquisition. The quantification of axon growth was performed by investigators masked to conditions. 2 layers (4 μm) at the depth of 1/4, 1/2 and 3/4 of the Z-stacks were selected for estimation of axon density of the optic nerve, the number of CTB positive axons that crossing perpendicular lines distal to the crush site at 250, 500 and 750 μm were counted, the widths of the nerve at each Z stack were measured as R and the radius (r) of the optic nerve was set from the widest R, to estimate the axon density in each depth: axon number/(R*4), the estimated axon number/optic nerve at each distances was then calculated by multiplying averaged density from three depths and cross-sectional area of axon ($\:A=\pi\:{r}^{2}$). CTB positive fibers that crossing perpendicular lines at 1000, 1500, 2000, 2500, 3000, 3500 and 4000 μm away from lesion site were counted on maximum projection images as the actual axon numbers of cleared optic nerves, the logarithm (base 10) of the actual axon counts was plotted, samples without axons were not displayed as dots. The axon counts of each distance from 1000 to 4000 μm were added for comparison.

### BaseScope and quantification of flag-positive signal

Longitudinal optic nerve cryosections (14 μm thickness) from 2 weeks after intravitreal injection, or 2 weeks after ONC, and brain cryosections (20 μm thickness) from 2 weeks after intravitreal injection were collected and processed for BaseScope assays. In situ hybridization to detect Flag-tagged transcript was performed using BaseScope Detection Reagent Kit v2 (Advanced Cell Diganostics, ACD/Bio-Techne, 323900) following the manufacturer’s instructions. A customized probe with 1ZZ pair was designed by ACD to detect the Flag epitope present in our AAV constructs to ensure specificity. Briefly, target retrieval was first carried out in RNAscope Target Retrieval Reagent at 98–100 °C, followed by endogenous peroxidase blocking and protease digestion (Protease IV) in a HybEZ oven (40 °C). The Flag probe or control probe (DapB-1ZZ) was hybridized at 40 °C, slides were washed in RNAscope Wash Buffer, then the signal was developed using the BaseScope V2 amplification cascade with Fast RED chromogen according to the kit protocol with minor adjustments. Sections were air-dried and mounted with BaseScope Mounting Medium. Images were taken under bright field using Keyence microscope (Keyence, Japan). For each section, the number of positive puncta was counted within defined regions of interest and normalized to area (dots per mm^2^). The density of Flag-positive puncta was compared across groups for multiple comparisons. At 2 weeks after ONC, the Flag-positive puncta counts that crossed 500–4000 μm from the crush site every 500 μm were performed in a masked fashion on longitudinal optic nerve sections. The density of Flag-positive puncta from each distance, and the average density of Flag-positive puncta across all distances were conducted for multiple comparison within groups.

### Statistical analyses

Most experiments showed sufficiently large effect sizes to easily justify sample sizes. For the in vitro data sets exhibiting high variability, additional repeats alongside controls were conducted to support statistical analysis. Results are presented as means ± standard error (SEM). GraphPad Prism was used for statistical analysis. Normality test was performed on each data set, one-way ANOVA, two-way ANOVA or Kruskal-Wallis test was performed to compare means of multiple groups for normally distributed or non-normally distributed data sets, respectively, two-tailed unpaired t-test or Mann-Whitney test was used for comparison between two groups for normally distributed or non-normally distributed data sets, respectively. P values of ≤ 0.05 were regarded as statistically significant.

## Results

### Different regional targeting 3‘UTRs present distinct reporter recovery patterns in distal growth cones

**Fig. 2:**
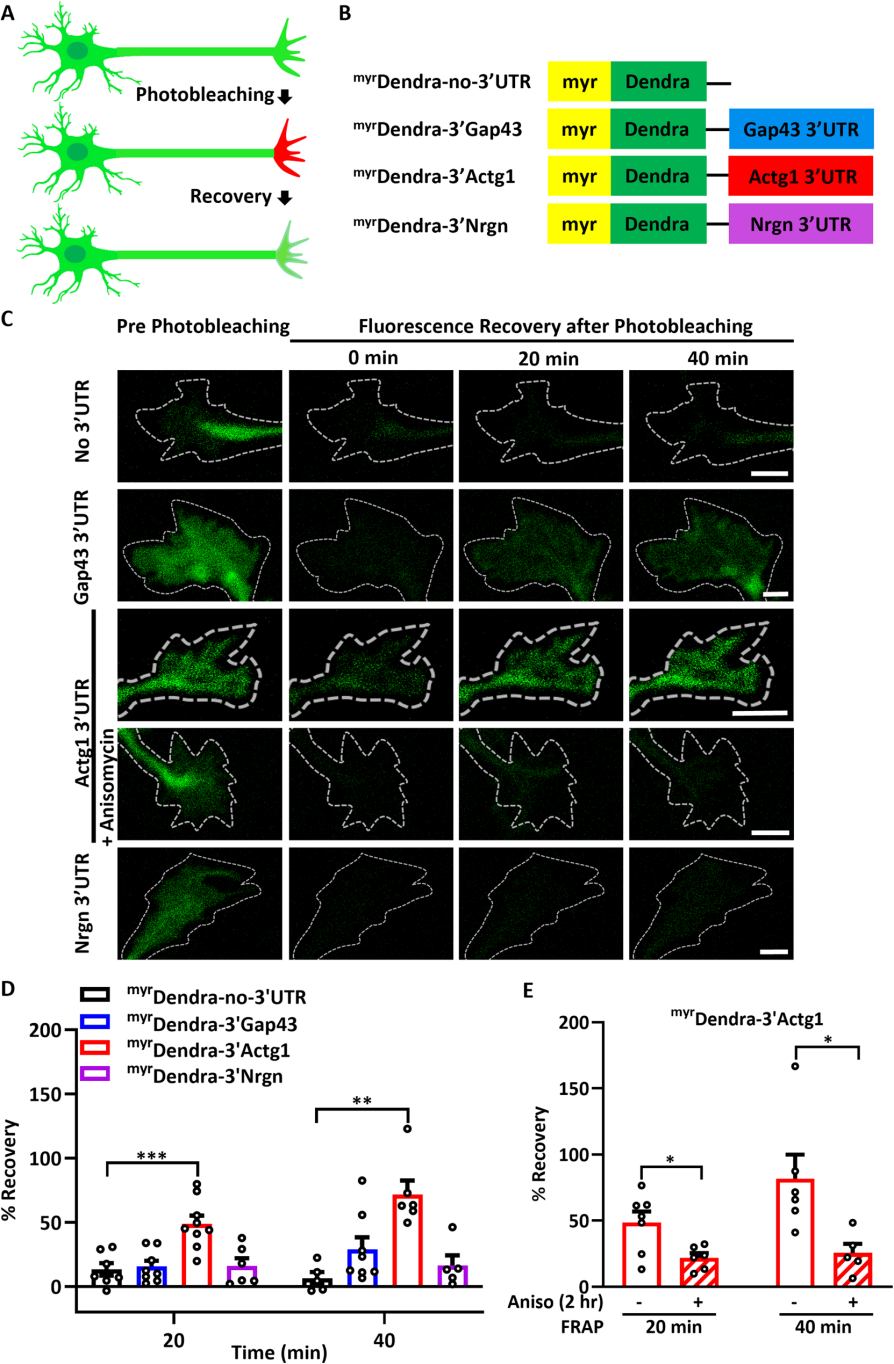
Growth cone translation is regulated differentially by 3’UTRs. (A) Schematic illustration of FRAP recording in distal growth cone of primary hippocampal neuron expressing photoswitchable fluorescent reporter gene myrDendra. (B) Diagram of different 3’UTR constructs with reporter myrDendra. (C) Representative images of FRAP in distal growth cones of hippocampal neurons expressing different myrDendra 3’UTR constructs 20 min and 40 min after photobleaching. In some cultures, anisomycin (20 μM) was applied to neurons transfected with myrDendra-3’Actg1 for 2 hr prior to FRAP recording. Growth cones were outlined with dashed lines. Scale bar, 5 μm. (D, E) Quantifications of FRAP as in (C). One-way ANOVA and Kruska-Wallis test were used in (D) (n=5-9 per group); two-tailed unpaired t-test was used in (E) (n=5-7 per group). All data shown as mean±SEM, *p<0.05, **p<0.01, ***p<0.001.

To explore RNA local translation and its dependence on 3’UTRs in distal axonal growth cones, FRAP recording was performed on primary hippocampal neurons (Fig. 2A), chosen for initial screening as they are also CNS neurons but easier to work with in generating in vitro transfected cultures. Constructs carrying photoswitchable fluorescent reporter Dendra fused with a diffusion-limited myristoylation element [36] were modified with various 3’UTRs, including axon-targeting Gap43 3’UTR [38], dendrite-targeting Nrgn 3’UTR [39], soma- and axon-targeting Actg1 3’UTR [35, 46], and a control construct lacking a 3’UTR (Fig. 2B), as we hypothesized that different UTRs, although they may distribute through the cell with overexpression, would still be able to demonstrate elements of UTR-specific regulation. As expected, ^myr^Dendra protein was detected everywhere within the neuron, but local photobleaching at growth cones decreased ^myr^Dendra’s green fluorescence and ^myr^Dendra fluorescence recovery was not seen in distal growth cones of neurons with the expression of ^myr^Dendra-3’Gap43, ^myr^Dendra-3’Nrgn, or no 3’UTR control (Fig. 2C, D), whereas significant recovery of Dendra signal was observed in neurons expressing soma- and axon-targeting ^myr^Dendra-3’Actg1 both at 20 min and 40 min (Fig. 2C, D). To distinguish whether the increase of Dendra is due to new local translation rather than from transport, we performed FRAP with the protein synthesis inhibitor anisomycin. In separate experiments, this Actg1 3’UTR-mediated fluorescence recovery was significantly inhibited by anisomycin (Fig. 2E), demonstrating that recovery was due to local new protein synthesis in the distal growth cone, rather than transport from the soma or nearby axon.

### Gap43 3’UTR-mediated translation is enhanced by neurotrophic factors and cAMP elevation

**Fig. 3:**
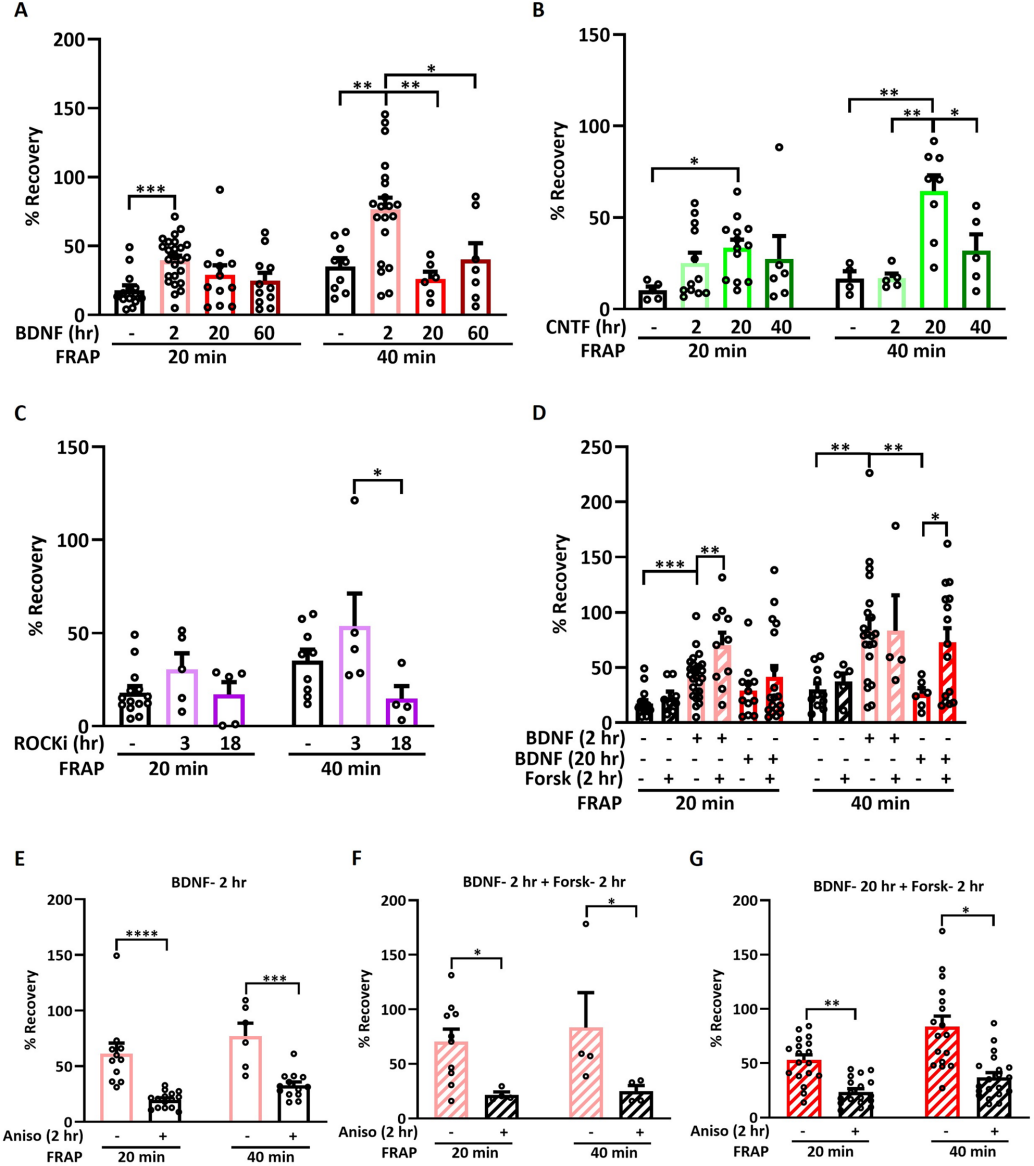
3’Gap43 UTR responds to neurotrophic factors and cAMP elevation. (**A-D**) FRAP quantifications of reporter ^myr^Dendra recovery in distal growth cones when ^myr^Dendra-3’Gap43-expressing hippocampal neurons were treated for times as marked with (**A**) BDNF, (**B**) CNTF, (**C**) ROCK inhibitor, or (**D**) BDNF with or without (data from **A**) forskolin. (**E-G**) Control experiments with anisomycin demonstrating that FRAP signal recovery is due to local translation in response to (**E**) BDNF or (**F, G**) BDNF and forskolin, at times in culture as marked. One-way ANOVA and Kruska-Wallis test were used in (**A, B**) (**A**, *n* = 6–25 per group; **B**, *n* = 4–13 per group); Kruska-Wallis test was used in (**C**) (*n* = 4–16 per group); Mann-Whitney and two-tailed unpaired *t*-test were used in (**D, E-G**) (**D**, *n* = 4–26 per group; **E**, *n* = 6–15 per group; **F**, *n* = 4–10 per group; **G**, *n* = 17–20 per group). All data shown as mean ± SEM, *p < 0.05, **p < 0.01, ***p < 0.001, ****p < 0.0001

BDNF, CNTF and Rho kinase inhibition (ROCKi) have been reported to promote RGC axon outgrowth in our previous studies [47, 48]. To explore whether these treatments affect local translation in distal growth cone, neurons transfected with ^myr^Dendra-3’Gap43 were treated with BDNF (Fig. 3A), CNTF (Fig. 3B), or the ROCKi Y-27,632 (Fig. 3C) for different durations. FRAP recovery in distal growth cones with ^myr^Dendra-3’Gap43 increased 20 and 40 min after photobleaching with a peak at 2 h treatment with BDNF that was lost by 20–60 h of BDNF (Fig. 3A). CNTF-induced FRAP peaked at 20 h (Fig. 3B). ROCKi did not show a difference from controls at 3 h and may have shown some decrease or toxicity at 18 h (Fig. 3C). The adenylate cyclase activator, forskolin, was next investigated as elevated intracellular cAMP enhances surface tropomyosin receptor kinase B (TrkB) expression [49]. Forskolin slightly increased FRAP response to 2 h BDNF at the 20 min time point; although 20 h of BDNF alone showed no effect, forskolin significantly increased Dendra recovery in response to 20 h of BDNF at the 40 min time point, even though forskolin alone had no effect (Fig. 3D; data in 3A and 3D are from the same experiments). Again, the new protein synthesis inhibitor anisomycin blocked the effects seen in conditions that showed significant reporter recovery (as in Fig. 3A and D), which were 2 h of BDNF, 2 h of BDNF plus forskolin, and 20 h BDNF plus 2 h of forskolin, at 20 min and 40 min after photobleaching (Fig. 3E, F, G), indicating the dependence of local protein translation on BDNF- or combined BDNF and forskolin-stimulated recovery of ^myr^Dendra-3’Gap43. Thus the 3’UTR of Gap43 confers no detectable growth cone-localized translation in the basal state, but enhances translation in response to neurotrophic factors and cAMP elevation.

### High baseline Actg1 3’UTR-mediated translation is further enhanced by cAMP elevation

**Fig. 4:**
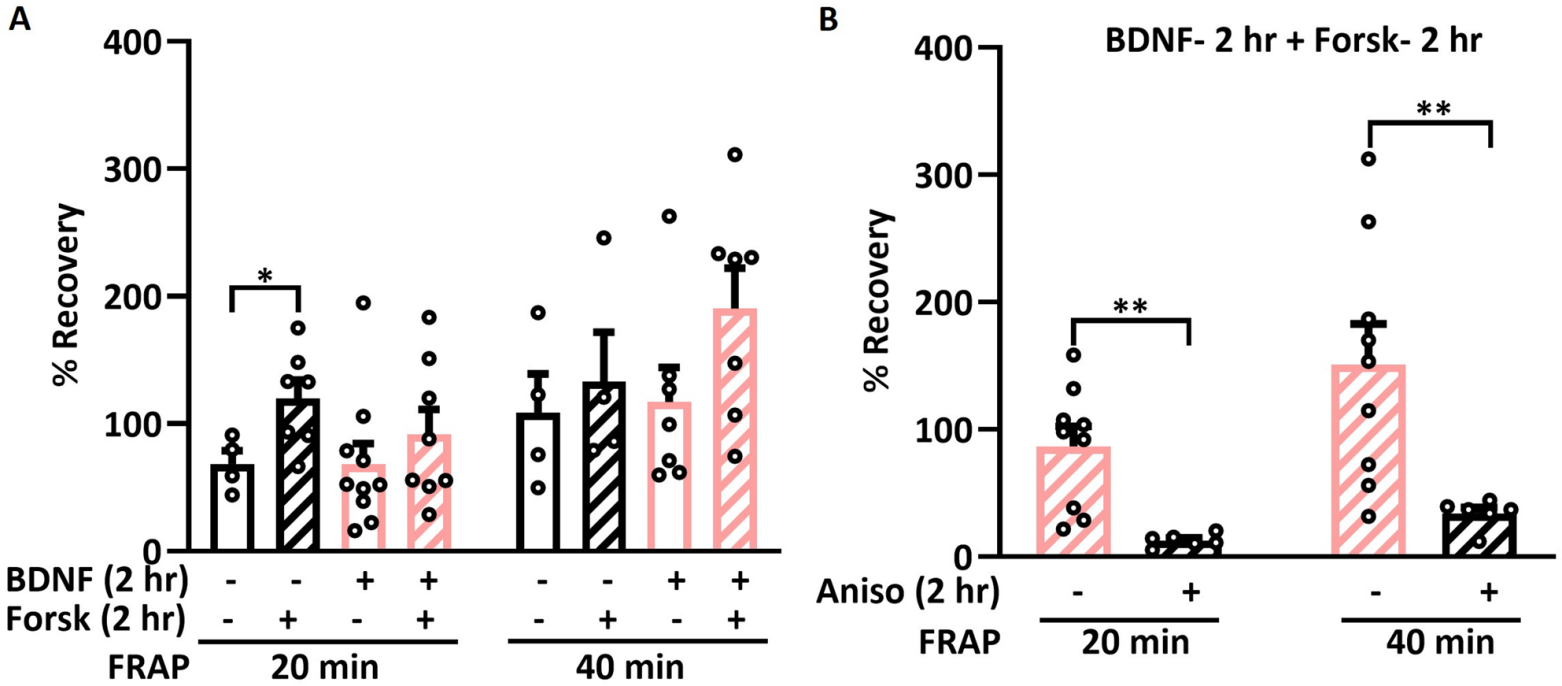
3’Actg1 UTR shows high basal local translation in growth cones with minimal response to BDNF or forskolin. (**A**,** B**) Quantification of FRAP analyses of reporter ^myr^Dendra-3’Actg1-expressing neurons with treatments as indicated does not show significant promotion of local translation in response to BDNF or forskolin (**A**), but confirms that all recovery is still due to local translation after treatment with anisomycin (**B**). Mann-Whitney and two-tailed unpaired *t*-test were used in (**A, B**) (**A**, *n* = 4–10 per group; **B**, *n* = 6–9 per group). All data shown as mean ± SEM, *p < 0.05, **p < 0.01

In parallel experiments, we found that ^myr^Dendra-3’Actg1, which showed a high basal level of growth cone FRAP (as in Fig. 2) showed little additional response to BDNF or forskolin, other than enhancement with forskolin alone detected at the 20 min time point (Fig. 4A). Dendra recovery to > 100% of baseline was observed in all groups at 40 min after photobleaching; however, there was no significant difference (Fig. 4A). The administration of anisomycin inhibited Dendra recovery of ^myr^Dendra-3’Actg1 with BDNF and forskolin (Fig. 4B), demonstrating that the Dendra recovery relied on local protein synthesis. These data taken together suggest that Actg1 3’UTR’s high basal level of local growth cone translation is not greatly affected by BNDF or cAMP signaling.

### Repolarization enhances local translation of reporter gene in growth cones with axon-targeting Gap43 3’UTR

**Fig. 5:**
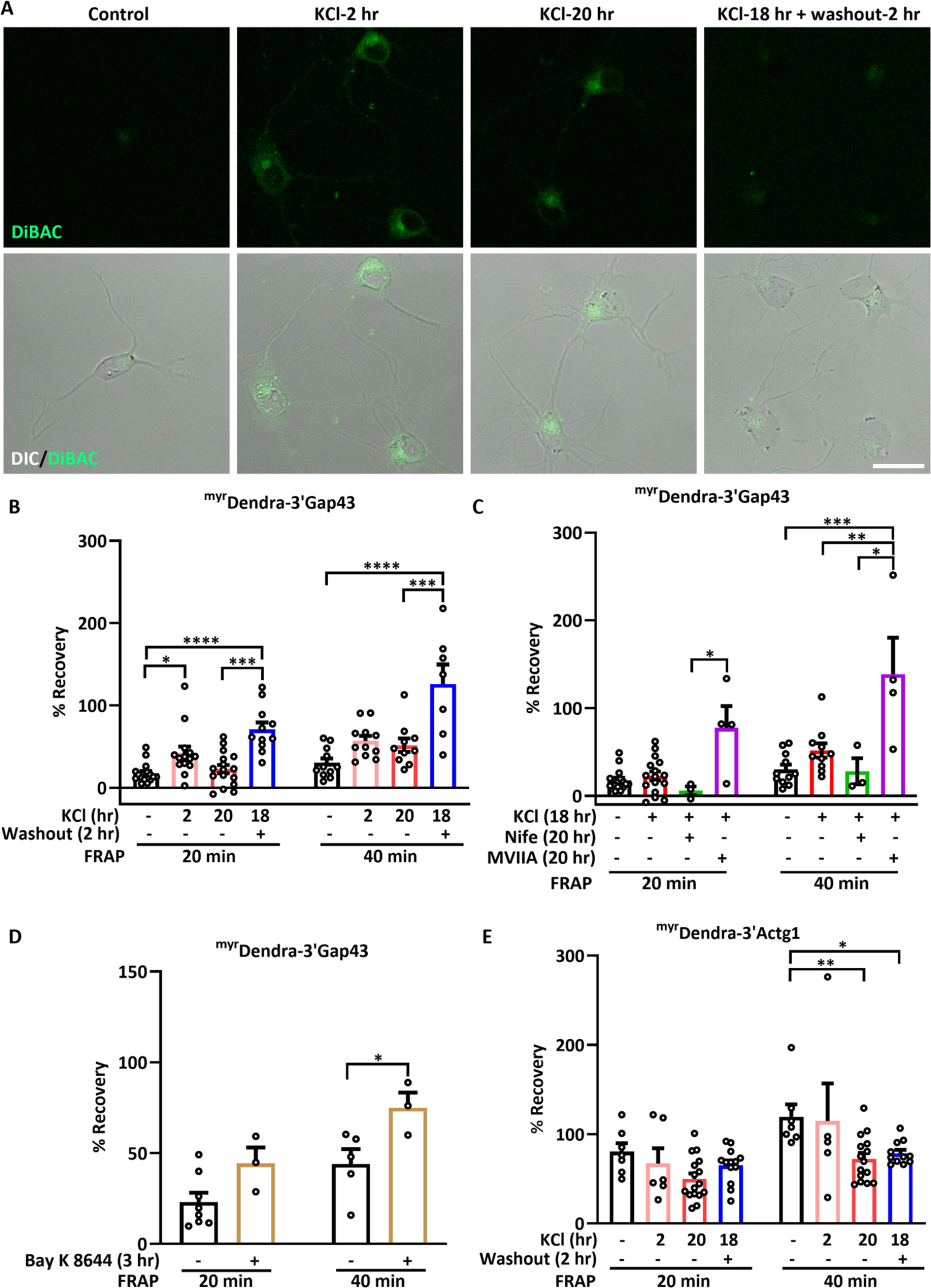
Local growth cone translation response to neuronal activity is 3’UTR-specific. (**A**) Neurons treated with KCl as marked were stained with depolarization-sensitive dye DiBAC_4_(3); fluorescent and DIC images were captured under live cell conditions. Scale bar, 20 μm. (**B-D**) Quantification of FRAP analyses of reporter ^myr^Dendra recovery in distal growth cone from neurons expressing ^myr^Dendra-3’Gap43 in response to (**B**) KCl with or without washout, (**C**) L-type calcium channel blocker nifedipine and N-type calcium channel blocker MVIIA, (**D**) calcium channel agonist Bay K 8644, at times in culture as indicated. (**E**) Quantification of FRAP analyses of reporter ^myr^Dendra-3’Actg1-expressing neurons with treatments of KCl with or without washout. Kruska-Wallis test and one-way ANOVA were used in (**B, C, E**) (**B**, *n* = 7–16 per group; **C**, *n* = 4–16 per group; **E**, *n* = 5–16 per group); two-tailed unpaired *t*-test was used in (**D**) (*n* = 3–8 per group). All data shown as mean ± SEM, *p < 0.05, ** *p* < 0.01, ***p < 0.001, ****p < 0.0001

Our previous findings revealed that neural activity induced by depolarization promotes axon outgrowth [47, 50], and depolarization of neurons triggered by KCl stimulation elevates cellular cAMP and surface TrkB expression [49, 51]. Here, we sought to detect the effect of neural activity on local protein translation in distal growth cones. Primary neurons transfected with ^myr^Dendra-3’Gap43 and ^myr^Dendra-3’Actg1 were subjected to FRAP following KCl-induced depolarization, or subsequent washout for repolarization, or calcium channel blockers or agonist. As indicated by the membrane potential-sensitive dye DiBAC_4_(3), intracellular fluorescence increased following both 2 h and 20 h of KCl treatment; this increase was attenuated after washout of KCl for 2 h (Fig. 5A). With the 3’Gap43 UTR, 2 h of depolarization induced a small increase in recovery not seen later at 20 h of depolarization, although the largest increase came with the re-hyperpolarization of washout after KCl (Fig. 5B). Interestingly, we found no significant effect on local translation with pre-treatment of L-type blocker nifedipine followed by KCl, whereas N-type blocker MVIIA significantly increased local translation at 40 min after photobleaching (Fig. 5C). We furthermore found that agonist Bay K 8644 which increases calcium entry through L-type channels increased distal growth cone translation at 40 min after photobleaching (Fig. 5D). Conversely, for the 3’Actg1 UTR which shows higher basal level of FRAP recovery, there was little effect of depolarization, although a suppression of basal growth cone translation was detected by 40 min after 18–20 h of KCl with or without washout (Fig. 5E). These results revealed that local translation in the growth cone especially for the low basal levels with 3’Gap43 UTR is regulated by neuronal activity.

### 3’UTRs regulate mRNA and protein distal axon localization and activity in vivo

**Fig. 6:**
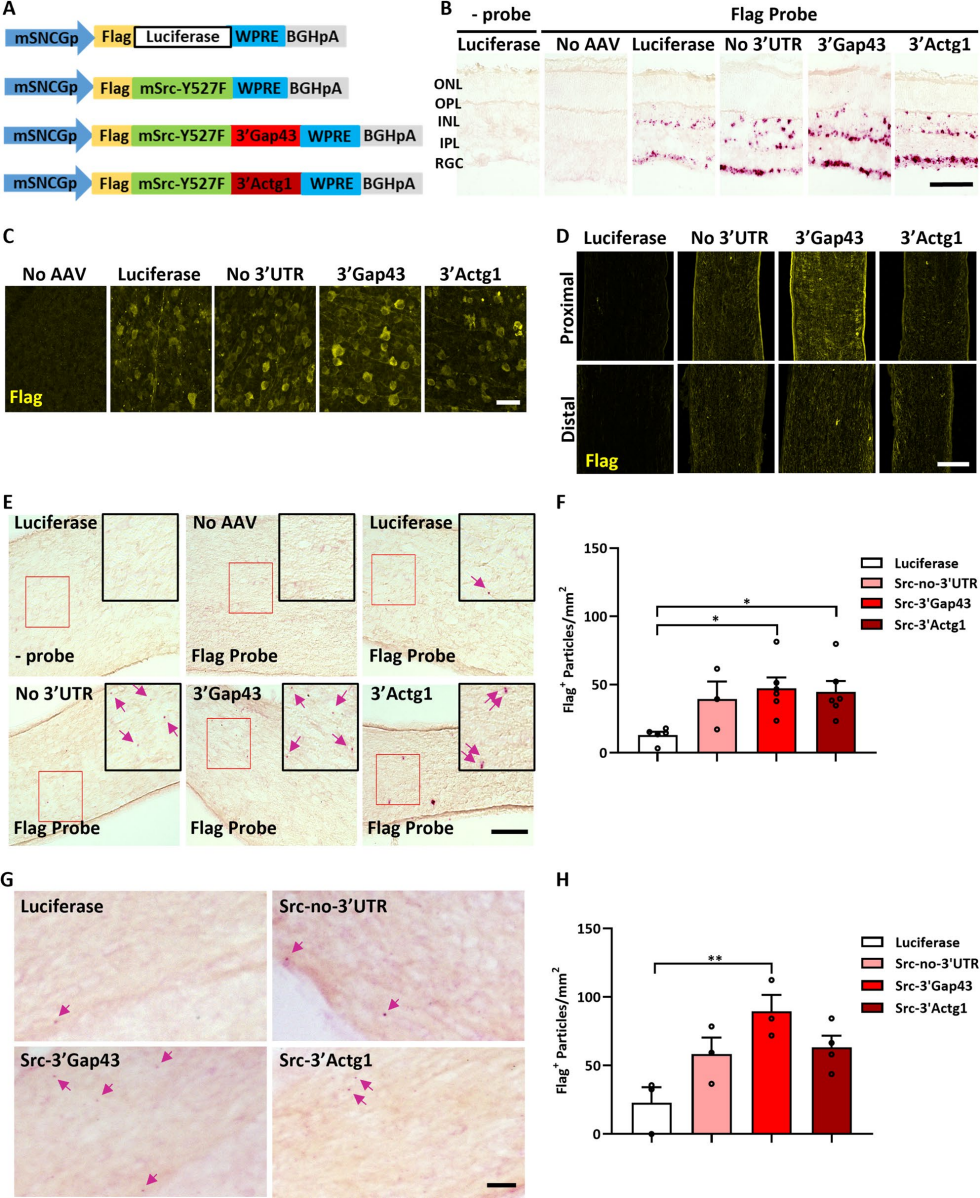
3’UTRs differentially drive distal Src mRNA and protein expression in the visual pathway. (**A**) Diagram of AAV constructs carrying luciferase and constitutively active Src with different 3’UTRs as indicated. (**B**,** E**,** G**) Representative images of BaseScope probe against Flag sequence (punctate red dots) in retina (**B**), optic nerve (**E**) and optic tract (**G**) (indicated by arrows) sections at 2 weeks after intravitreal injection of AAVs, with a no-AAV control and a negative control probe (DapB) (selected from AAV-luciferase-treated eyes). Scale bar, 100 μm in (**B**), 50 μm in (**E**), 20 μm in (**G**). (**C**,** D**) Representative confocal images at 2 weeks after intravitreal injection of AAVs of (**C**) flatmounted retina or (**D**) proximal and distal optic nerves showing Flag-positive cells and axons, respectively. Scale bar, 50 μm in (**C**), 100 μm in (**D**). (**F**,** H**) Quantification of the density of Flag-positive spots in (**E**) optic nerve and (**G**) optic tract. Kruskal-Wallis test was used for multiple comparison in (**F**) (*n* = 3–6 per group), one-way ANOVA was used for multiple comparison in (**H**) (*n* = 3–4 per group). All data shown as mean ± SEM, *p < 0.05, ** *p* < 0.01

We next asked whether these 3’UTRs direct RNA localization in vivo. To address this question, we selected a previously studied growth cone protein Src [52, 53], we then designed AAVs to express constitutively active Src with different 3’UTRs, so as to identify the impact of 3’UTRs in traumatic optic nerve injury model. We generated AAV constructs with mSncg promoters for RGC-specific expression [40] to express Flag-tagged, constitutively active Src with 3’Gap43, 3’Actg1 or no 3’UTRs; AAV-luciferase was used as a negative control (Fig. 6A). AAVs were intravitreally injected 2 weeks before tissue harvest. A 1ZZ BaseScope Flag probe detected positive Flag RNA expression in the ganglion cell layer and to a lesser degree inner nuclear layer of the retina after transduction with AAVs, not seen with a negative control probe or retinal sections with no AAV (Fig. 6B). Flag mRNA was detected much less frequently in optic nerve (Fig. 6E) and optic tract (Fig. 6G) sections than in retina, but quantification showed a significantly higher density in the optic nerve tissues treated with Src-3’Gap43 or Src-3’Actg1- (Fig. 6F), as well as in Src-3’Gap43-transduced optic tract sections (Fig. 6H), compared to the luciferase control group. At the protein level, Flag tag was detected in all AAV-injected retina flatmounts (Fig. 6C) and in the proximal and distal optic nerves (Fig. 6D).

**Fig. 7:**
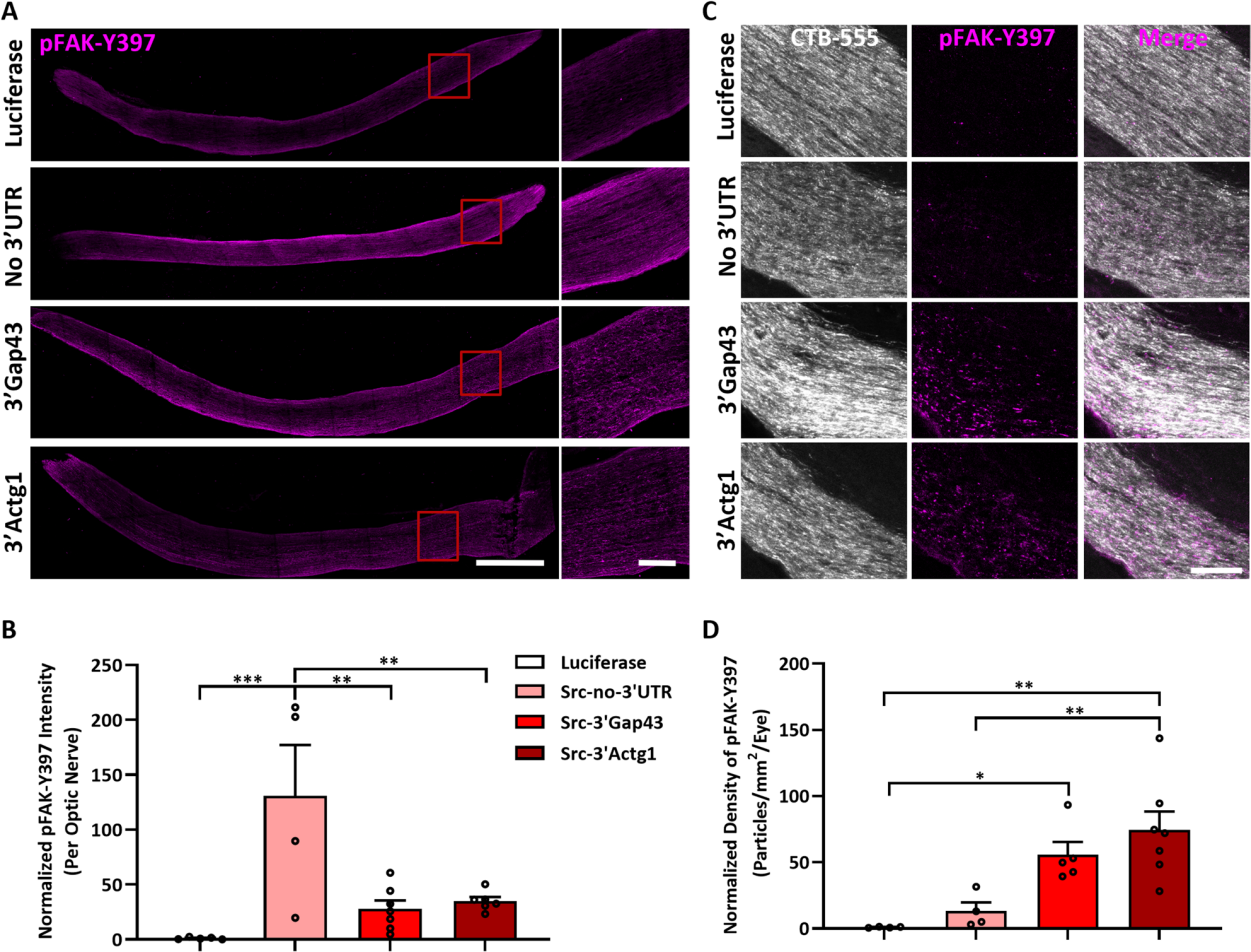
3’UTRs differentially drive enzymatic activity in the visual pathway. (**A**) Phospho-FAK-Tyr397 detection in optic nerves, including higher magnified images of indicated areas close to the chiasm in the right panels. Scale bar, 500 μm in left panels and 100 μm in right panels. (**B**) Quantification of data from (**A**), normalized to luciferase controls. (**C**) Phospho-FAK-Tyr397 detection in optic tracts, confirmed with CTB555 anterograde labeling of RGC axons, at 2 weeks after intravitreal injection of AAVs as marked. Scale bar, 50 μm. (**D**) Quantification of data from (**C**), normalized to luciferase controls. One-way ANOVA was used for multiple comparisons in (**B, D**) (*n* = 4–7 per group). All data shown as mean ± SEM, *p < 0.05, ** *p* < 0.01, *** *p* < 0.001

Activated Src triggers phosphorylation of downstream kinase FAK at Y397 [54]. In the uninjured visual system, we detected significantly more pFAK Y397 in the Src-no-3’UTR construct in the optic nerve than other groups (Fig. 7A, B). More pFAK Y397 was detected in the more distal optic tract (identified by anterograde transport of CTB555) after transduction with Src-3’Gap43 or Src-3’Actg1 than luciferase (Fig. 7C, D). The difference between 3’Gap43 and 3’Actg1 UTRs was not significant, more pFAK significance was indicated from optic tract with 3’Actg1 UTR than with Src-no-3’UTR (Fig. 7D).

These results suggest that the axon-targeting 3’Gap43 UTR and the broadly targeting 3’Actg1 UTR facilitate increasingly distal axon localization and detectable differences in Src mRNA (as detected by in situ hybridization) and Src activity (as detected by pFAK) in optic nerve axons and optic tracts in vivo.

### Src with an axon-targeting 3’UTR promotes RGC regeneration

**Fig. 8:**
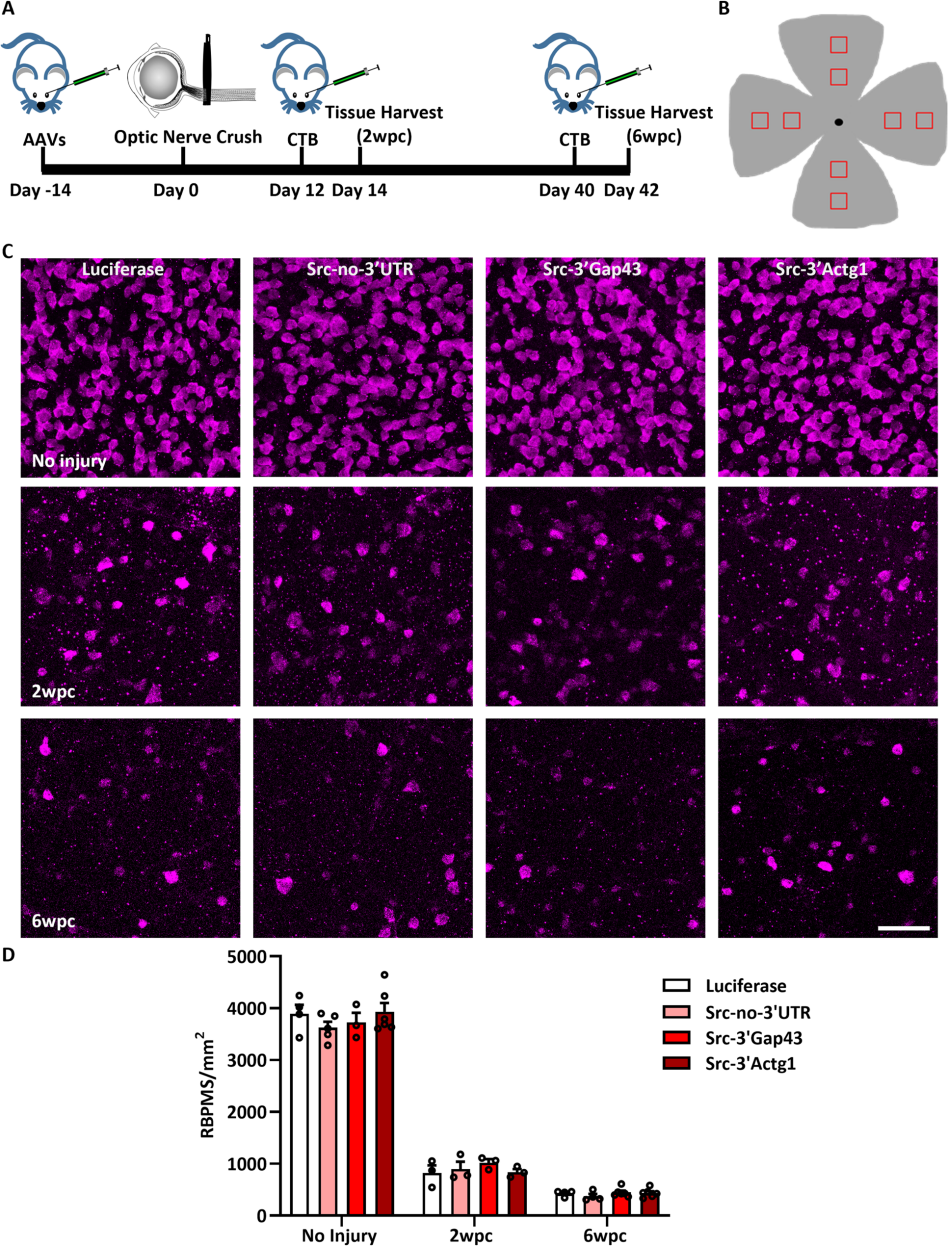
Src expression does not increase RGC survival after optic nerve injury. (**A**) Schematic pipeline of experimental design. (**B**) Eight areas from middle and peripheral retinal flatmounts as illustrated were imaged and quantified. (**C**) Representative images of RBPMS-positive RGCs from AAV-transduced retinas with no injury, or 2 or 6 weeks after optic nerve crush. Scale bar, 50 μm. (**D**) Quantification of RBPMS-positive RGCs counting from different groups as in (**C**). One-way ANOVA and Kruska-Wallis test were used in (**D**) (*n* = 3–6 retinas per group). All data shown as mean ± SEM

Src has been reported to support peripheral neuron survival and axon regeneration in previous studies of dorsal root ganglion neurons [55]. Here we assessed the effect of Src with 3’Gap43, 3’Actg1 or no 3’UTRs on CNS RGC survival and optic nerve regeneration after optic nerve injury (Fig. 8A). RGC survival from 2 to 6 weeks after optic nerve crush injury (2wpc, 6wpc) was quantified in central and peripheral retina by RGC-specific marker RBPMS immunostaining (Fig. 8B). Optic nerve crush induced ~ 75% of RGC death at 2wpc, and more than 85% at 6wpc, and constitutively active Src exhibited no significant effect on RGC survival regardless of 3’UTR (Fig. 8C, D).

**Fig. 9:**
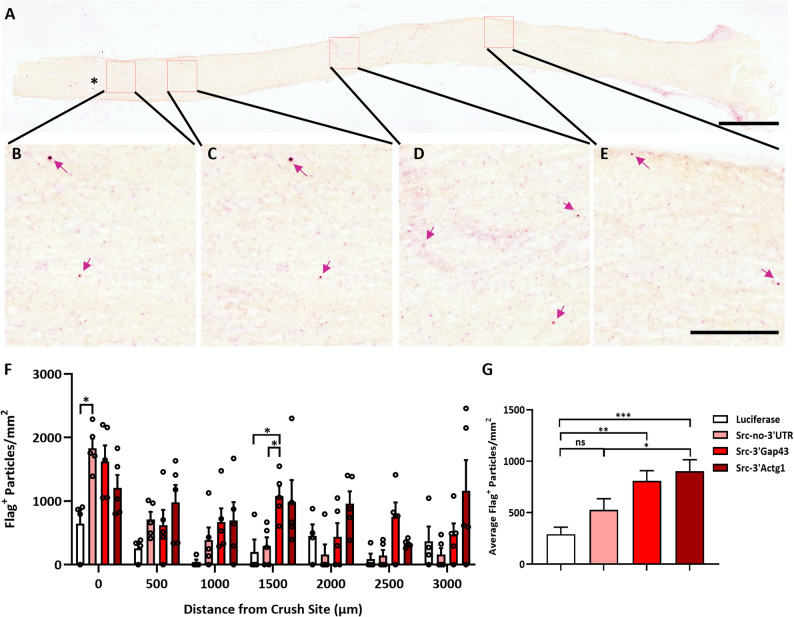
3’UTRs increase Src mRNA in optic nerve after injury. (**A**), Representative images of BaseScope probe against Flag sequence (punctate red dots) in optic nerve sections transduced with Src-3’Gap43 at 2 weeks after optic nerve crush (scale bar, 500 μm), crush site indicated with *. (**B-E**) Magnified BaseScope images of optic nerve areas as indicated in (**A**), showing example Sects.  0–500 μm (**B**), 500–1000 μm (**C**), 1500–2000 μm (**D**), and 2500–3000 μm (**E**) from the crush site. Scale bar, 100 μm. (**F**,** G**) Quantification of Flag-positive puncta density at each distance from the lesion site (**F**), and average density of Flag-positive puncta across all distances (**G**). Two-way ANOVA was used in (**F**), Kruskal-Wallis test was used for multiple comparisons in (**G**) (*n* = 4–5 per group). All data shown as mean ± SEM, *p < 0.05, ** *p* < 0.01, ***p < 0.001

We then asked whether target mRNAs could be detected in the optic nerve after injury. We conducted BaseScope against Flag probe to evaluate the effect of Src with 3’Gap43, 3’Actg1 or no 3’UTRs on Flag mRNA expression, now showing BaseScope of Flag across the full optic nerve treated with Src-3’Gap43 (Fig. 9A), along with magnified images from various distances from the crush site (Fig. 9B-E). At 2wpc we detected more positive Flag RNA expression in the optic nerve, reaching statistical significance for any individual distance at 1500 μm from the crush site in Src-3’Gap43-treated tissue than in the Src-no-3’UTR and luciferase groups (Fig. 9F), but when analyzed in aggregate across the full length of analyzed optic nerve, the average density of positive Flag puncta after Src-3’Gap43 and Src-3’Actg1 were significantly increased compared to in Src-no-3’UTR-treated tissue and compared with the luciferase control group (Fig. 9G). Thus 3’UTRs from Gap43 and Actg1 increases optic nerve localization of the exogenous Src mRNA.

**Fig. 10:**
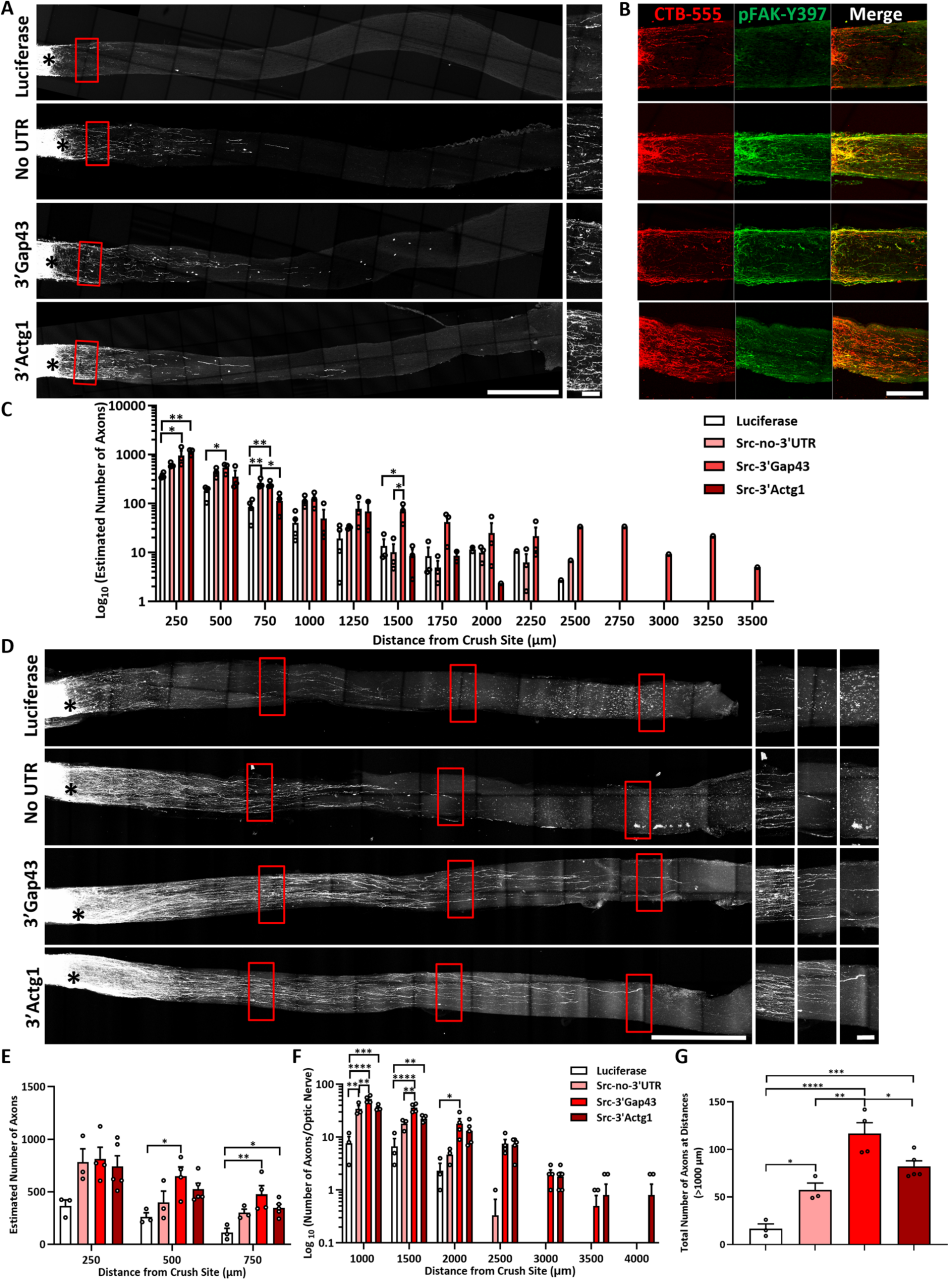
Src promotion of RGC axon regeneration is increased with 3’Gap43 UTR. (**A**), Representative confocal images of optic nerve sections after anterograde CTB555 labeling at 2 weeks after optic nerve crush (left panels; scale bar, 500 μm), crush site indicated with *. Right panels show magnified images of each sample from 250 to 350 μm away from crush site as marked; scale bar, 50 μm. (**B**) Optic nerve sections were labeled with CTB555 and pFAK-Y397 at 2 weeks after optic nerve crush, images showing 0–250 μm from crush site. Scale bar, 50 μm. (**C**) Quantification of regenerating axons as in (**A**) shows 3’Gap43 UTR, and at shorter distances also 3’Actg1 UTR, further enhance Src-promoted axon regeneration. (**D**) At 6 weeks after optic nerve crush, representative confocal images of optic nerve wholemounts after iDISCO clearing (left panel scale bar, 500 μm) and magnified insets from 1000–1100 μm, 2000–2100 μm and 3000–3100 μm from lesion site (right panels scale bar, 50 μm). (**F-G**) Quantification of regeneration at 6 weeks using (**E**) estimated axon numbers per optic nerve at 250 μm, 500 μm and 750 μm from lesion site, (**F**) actual number of axons at distances at or longer than 1000 μm from lesion site, and (**G**) total number of axons per treatment group from 1000–4000 μm from lesion site. One-way ANOVA and Kruska-Wallis test were used (**C, E-F**) (*n* = 3–5 optic nerves per group). All data shown as mean ± SEM, *p < 0.05, ** *p* < 0.01, ***p < 0.001, ****p < 0.001

In contrast, at 2wpc we detected significantly more short-distance axon sprouting and long-distance axon regeneration after Src-3’Gap43, reaching statistical significance at 250 μm, 500 μm, 750 μm and 1500 μm, and continuing to trend towards significance at even longer distances (Fig. 10A, C). Src-3’Actg1 also showed long distance axon regeneration even past 2500 μm to the optic chiasm in one sample (Fig. 10C). The activation of Src target pFAK-Y397 was identified in CTB555-positive regenerating axons in all Src groups but not in the occasionally detected regenerating axons in the luciferase controls (Fig. 10B), consistent with Src-induced FAK activation supporting long-distance axon regeneration.

At 6wpc both Src-3’Gap43 and Src-3’Actg1 significantly increased long-distance axon regeneration, reaching statistical significance at a variety of shorter (Fig. 10D, E) and longer (Fig. 10F) distances. Note that small puncta of fluorescence (e.g. see past 3000 μm, Fig. 10D) were not counted as indicative of axons, as these could be attributable to degenerative changes. Cumulative axon counts at any distance from 1000 μm revealed significantly higher axon regeneration in any Src group compared to luciferase, with Src-3’Gap43 showing a significant advantage over either Src-3’Actg1 or Src-no-3’UTR, which did not differ from each other (Fig. 10G). Taken together, these findings indicate that constitutively active Src expression promotes axon regeneration, but even more robustly with the addition of the axon-targeting 3’Gap43 UTR.

## Discussion

Taken together, these data significantly extend our understanding of 3’UTRs’ regulation of translation in growth cones, including in response to internal and external signals, and demonstrate the premise of leveraging 3’UTRs to promote axon regeneration after injury in the CNS. Considerable prior work has identified many mRNAs localized in axons, and distinct mRNA translatomes are detected in developing versus adult and CNS versus PNS axons [20, 31]. For example, developing RGC axons and to some degree embryonic peripheral dorsal root ganglia (DRG) axons localize mRNAs implicated in axon growth and guidance and synaptogenesis, whereas adult RGC axon mRNAs are linked to axonal survival and neurotransmission [20, 31]. However, little work has followed to understand how these mRNAs may be differentially regulated in translation in the axon or growth cone. Here we develop this work with 3 significant advances.

First, using a novel in vitro FRAP platform in growth cones of primary neurons and testing 3’UTRs that have been reported to direct localizations of mRNAs to axons (3’Gap43 UTR), soma and axons (3’Actg1 UTR), dendrites (3’Nrgn UTR), or no 3’UTR as a negative control, we characterized local growth cone translation. To explore local protein synthesis within growth cones or other neuronal sub-compartments, FRAP has been applied in DRGs and motoneurons, using a reporter GFP with a myristylation signal for membrane targeting [46, 56]. Here, we established FRAP to monitor dynamic translation by upgrading to photoswitchable Dendra with a myristylation sequence in primary CNS neurons in vitro. In this system, pre-existing proteins were efficiently and irreversibly photoconverted into a red fluorescent form, and newly synthesized proteins were visualized by green fluorescent signal [37], enabling precise and stable tracking of regional translation in living cells. Additionally, the administration of protein synthesis inhibitor anisomycin provided a critical control to confirm that the observed recovery signals relied on local new translation rather than protein transport, or protein transport of a myristoylated membrane-bound protein, for that matter. Our results displayed distinct reporter recovery patterns in growth cones with soma/axonal 3’Actg1, axonal 3’Gap43, dendritic 3’Nrgn and control no 3’UTR constructs, in which only neurons expressing 3’Actg1 UTR displayed a high basal level of local translation in the growth cone. Thus this model already argued for the relevance of 3’UTRs in regulated local protein synthesis in distal growth cones of CNS neurons.

Second, this model allowed for the unveiling of differences in the signaling regulation of local translation dependent on 3’UTRs. Focusing on 3’Gap43 and 3’Actg1, we characterized recovery dynamics in response to the neurotrophic factors BDNF and CNTF, adenylate cyclase activator forskolin, cytoskeletal regulation with ROCKi, and KCl-induced depolarization, washout-mediated repolarization, and calcium channel blockers or agonist. Interestingly, the axon-specific Gap43 3’UTR demonstrated a lower basal level of growth cone translation, but was more responsive to stimulation with neurotrophic factors or cAMP, or to re-hyperpolarization after washout of KCl, or to pre-treatment with N-type blocker MVIIA followed by KCl-induced depolarization. In contrast, 3’Actg1 UTR only showed significant elevation induced by forskolin. We and others previously reported the effects of BDNF, CNTF, forskolin, and depolarization on RGC survival and axon growth in vitro [47, 57] and in vivo [58, 59]. These may act through pathways impacting many aspects of neuronal biology to promote axon growth [2, 50, 60] and our data now suggest another potential pathway, namely local regulation of mRNA translation in axons or growth cones. In particular, our data suggest that local translation can be differentially regulated by specific UTRs, as for example 3’Gap43 UTR increased but 3’Actg1 UTR suppressed local translation in response depolarization and re-hyperpolarization. Future work will be directed to dissect pathways and how the signaling downstream impact local translation.

How 3’UTRs regulate local translation and respond to local signals is likely attributable to RNA-binding proteins (RBPs). Highly conserved and broadly distributed across tissues, RBPs target a wide range of RNA molecules such as mRNAs, rRNAs, tRNAs, snRNAs and other ncRNAs [61], and RBPs are critical in regulating transport, localization, splicing, stability, and translation of bound RNAs, all of which are essential to neuronal function in neurodevelopment and neurodegenerative diseases [62]. More than 3000 human RBPs have been reported in experimental and annotation data sets [61, 63], and recognize and bind specific RNAs via RNA-binding domains or motifs such as RNA recognition motif (RRM), K-homology (KH) domain, zinc-finger domain, and around 30 other domains [64]. Different RBP families have been categorized based on their RNA-binding domains or cellular functions in the CNS, including human antigen D (HuD) [65], CUGBP Elav-like (CELF) [66], heterogeneous nuclear ribonucleoprotein family (hnRNP) [67], fragile X mental retardation protein (FMRP) [62], etc. The HuD protein interacts with AU-rich element (ARE) in 3’UTR of target RNAs including Gap43 UTR studied here, Tau, and Neuritin1(Nrn1) [68–71], and HuD is capable of localizing ARE-containing mRNA into axons [38]. No RBP for Actg1 3’UTR has been reported yet. RBPs are likely critical for mediating many axon transport-associated functions; for example, RGC survival after optic nerve injury in vivo depends on the anterograde transport motor protein kif5a and also on kif5a’s associated RBP HnRNPA1 [72]. Future research should explore how RBPs target specific motifs in 3’UTRs for stabilizing mRNA, transporting mRNA actively, and/or directing mRNAs to distinct subcellular locations, and how signaling pathways regulate RBP’s handling of their target mRNAs.

Third, we found that constitutively active Src modified with axonal 3’UTRs and particularly with the 3’Gap43 UTR significantly promoted long distance axon regeneration following optic nerve injury. The Src family kinases (SFK) are nonreceptor tyrosine kinases [73, 74] originally described to play regulatory roles in cell adhesion, proliferation, and survival during tumor development [75]. Subsequently, several lines of evidence implicated Src in modulating growth cone adhesion and actin dynamics in vitro [76] and in PNS axon regeneration in DRG neurons [55]. Src targets at the cytoskeleton include FAK [54] and others, but it was not known if Src could be preferentially targeted to distal axons of CNS neurons in vivo, towards further promoting axon regenerative responses. Here, using AAV2-mediated overexpression of constitutively active Src with axon-targeting 3’UTRs in RGCs, these data provide direct evidence for Src, and even more so Src with the axon-targeting 3’Gap43 UTR, in promoting axon regeneration at 2 weeks and 6 weeks after optic nerve injury in the CNS, providing valuable in vivo data to help confirm the proposition of regulatory 3’UTRs coming from in vitro. Future work will test longer time points and may explore whether other exogenous agents studied in vitro, like BDNF or cAMP analogues, could enhance the effects of Src with specific UTRs in promoting axon regeneration even further, experiments we did not explore here as local optic nerve/axon delivery needs to be solved for. Molecular evidence of distal axon transport of the mRNA, protein, and even its activity in the form of activation of pFAK-Y397, supported the model of 3’UTR amplification of Src mRNA transport and translation. These data do not distinguish whether there is also a significant role for Src in the cell body, as it was not possible to exclude the exogenous Src from the soma. Nevertheless, taken together these data support the premise of leveraging 3’UTR biology in further studies to generate therapeutic strategies for CNS regeneration.

Other limitations or areas for future work include that we used primary embryonic hippocampal neuron for in vitro study, as they are CNS neurons with a polarized morphology similar to adult RGCs but easier to work with in larger numbers. In vivo, the sparse labeling with BaseScope can limit interpretation, and the fact that we only studied Src with these UTRs instead of a host of other coding sequences may also limit generalizability, as some of the effects of Src localization may be due to the UTRs but may also be due to sequences in the Src coding mRNA. There may also be effects of overexpressed UTRs interfering with processing of UTRs, in a dominant-negative fashion for example. We didn’t explore the effect of control AAVs with 3’UTRs alone or coupled to luciferase on RGC survival or axon regeneration after optic nerve injury in vivo, but the sparse expression of the UTRs measured by BaseScope suggests that this is not a likely source of confounding.

## Conclusions

Finally, growth cone biology is clearly critical for axon growth, regeneration, and guidance [2, 16, 23, 77, 78]. Although some molecular manipulations that promote axon regeneration target axon transport, e.g. via kinesins [15, 72, 79], limited research has focused on translating our understanding of growth cone biology into therapeutic candidates, other than through regulation of growth cone dynamics [48, 80, 81]. Here bringing together the biology of growth cone cytoskeletal dynamics and the biology of local translation in growth cones points to the importance of focusing future work at this interface.

## Data Availability

No datasets were generated or analysed during the current study.

## Abbreviations

CNS: Central nervous system
3’UTRs: 3’ untranslated regions
FRAP: Fluorescence recovery after photobleaching
Actg1: Gamma-actin
BDNF: Brain-derived neurotrophic factor
CNTF: Ciliary neurotrophic factor
AAV: Adeno-associated viral
RGCs: Retinal ganglion cells
cAMP: Cyclic adenosine monophosphate
PNS: Peripheral nerve system
PBS: Phosphate-buffered saline
ROI: Regions of interest
ROCKi: Rho-associated kinase inhibitor
ONC: Optic nerve crush
PFA: Paraformaldehyde
OCT: Optimal cutting temperature
FAK: Focal Adhesion Kinase
RBPMS: RNA-binding protein with multiple splicing
DCM: Dichloromethane
DBE: Dibenzyl ether
SEM: Standard error
